# Doxorubicin-fucoidan-gold nanoparticles composite for dual-chemo-photothermal treatment on eye tumors

**DOI:** 10.18632/oncotarget.23092

**Published:** 2017-12-09

**Authors:** Hyejin Kim, Van Phuc Nguyen, Panchanathan Manivasagan, Min Jung Jung, Sung Won Kim, Junghwan Oh, Hyun Wook Kang

**Affiliations:** ^1^ Interdisciplinary Program of Biomedical Mechanical and Electrical Engineering, Pukyong National University, Busan, South Korea; ^2^ Department of Biomedical Engineering and Center for Marine-Integrated Biomedical Technology (BK 21 Plus), Pukyong National University, Busan, South Korea; ^3^ Department of Pathology, Kosin University College of Medicine, Busan, South Korea; ^4^ Department of Otolaryngology-Head and Neck Surgery, Kosin University College of Medicine, Busan, South Korea

**Keywords:** doxorubicin, gold-nanoparticles, photothermal therapy, chemotherapy, eye tumor

## Abstract

The current research demonstrates the feasible biomedical application of AuNPs coated with doxorubicin (Dox)-loaded fucoidan (Fu) for dual-chemotherapy and photothermal treatment (PTT) on eye tumors *in vitro* and *in vivo*. Marine-derived Fu was used as a capping agent to achieve high photostability for AuNPs, and Dox as a FDA-approved anti-cancer drug was added to induce chemotherapy. The synthesized Dox-Fu@AuNPs exhibited high cytotoxicity on the tumor cells and strong light absorption for temperature increase *in vitro*. After intratumoral injection of Dox-Fu@AuNPs in the rabbit eye tumors, PTT-assisted Dox-Fu@AuNPs entailed the complete removal of the eye tumors without recurrence for 14 days after the treatment. Photoacoustic image contrast from the tumor regions was enhanced due to selective light absorption by the administered Dox-Fu@AuNPs. Therefore, the proposed Dox-Fu@AuNPs can be a potential nano-theranostic material for treating and diagnosing the eye tumors.

## INTRODUCTION

Choroidal melanoma is the most common primary intra-ocular malignant tumor, and the incidence rate is approximately 6 per million in the United States [1]. The melanoma appears as a brown elevated dome-shaped tumor occurring in the sub-retinal space, and the malignant choroidal melanoma can metastasize to other parts of the body such as liver. Although the eye tumor is typically asymptomatic, the development of the choroidal melanoma can cause irregular astigmatism (blurred vision), retinal detachment along with decreased visual acuity, and even glaucoma with permanent vision loss. Over the last few decades, a great number of eye tumor treatments have been introduced, such as surgical incision, electromagnetic wave radiation, radiotherapy, hyperthermia, and photothermal treatment (PTT) [2–7]. However, the current treatments of the eye tumors still suffer from infection, invasive or ionizing natures, unpredictable thermal injury, and tumor recurrence [8]. Therefore, the alternative approach is still required to enhance the efficacy and safety of the eye tumor treatment even in a minimally invasive manner.

Nanomaterial-based PTT has extensively been investigated as a non-invasive or minimally invasive and effective therapeutic technique to treat various types of tumors *in vitro* and *in vivo* [9–13]. The primary purpose of this method is to employ light-absorbing agents and to locally generate heat upon light absorption, leading to irreversible thermal injury selectively to the targeted tumors. The nanomaterials have attracted an increasing interest in the biomedical field due to their facile synthesis and unique physicochemical properties. In addition, various nano-agents to enhance PTT efficacy have widely been investigated such as copper sulfide and golden carbon-based materials [11, 14, 15]. Among the potential photothermally active nanomaterials, gold nanoparticles (AuNPs) have been studied extensively for drug delivery, cellular/tissue imaging, and thermal therapy on account of strong optical coupling, extraordinary photon-to-thermal energy conversion efficiency, and easy functionalization [16–18]. In addition, Fucoidan (Fu) has been investigated as a chemo-preventive substance for nanomedicine due to their antitumor and anti-inflammation effects that suppress the tumor growth as well as significantly reduce the inflammation periods [19, 20]. Fu has often been employed for coating AuNPs to reduce the toxicity of the metallic nanoparticles [21–23]. However, *in vivo* applications of the Fu-coated AuNPs have yet been explored in terms of surface functionalization and therapeutic effects particularly on the eye tumors. Recently, chemotherapy drugs have also been encapsulated or conjugated with AuNPs to enhance PTT efficacy and to minimize tumor recurrence [24]. Doxorubicin (Dox) is a common chemotherapeutic agent that is used to treat human malignancies by inhibiting nucleic acid synthesis [22, 24–26]. Several researchers have suggested the potential use of polymers as drug carriers to maximize the efficacy of Dox by limiting toxicity [27]. However, the current Dox chemotherapy still have the primary limitations including administration of large volume, resultant high toxicity on normal healthy cells, and a short lifetime in the body [28]. Therefore, conjugation with hydrophilic polymers to overcome non-specificity and toxicity of Dox have been researched [29]. Moreover, as most nanomaterial-based PTT has been performed on the small-sized mice, further evaluations with larger animals are still required for clinical translation of the proposed technique. In fact, the large animals can be investigated effectively for the targeted therapy due to their larger tissue volume [30]. Therefore, to achieve synergistic cancer treatment with both thermal necrosis of PTT and chemotherapeutic effect by loaded Dox, gold nanoparticles were synthesized in the presence of Fu and were subsequently adsorbed by Dox. Due to anti-tumor and anti-inflammation effects as well as biocompatibility, Fu was selected to conjugate Dox with AuNPs by means of electrostatic physisorption interactions between positively charged groups (AuNPs and Dox) and negatively charged sulfate groups (Fu). The aim of the current study was to investigate the feasible application of Dox-conjugated Fu-encapsulated gold nanoparticles (Dox-Fu@AuNPs) in VX2 cells and xenograft tumors in mid-sized rabbits as an anti-cancer theranostic agent for effective eye tumor management (Supplementary Figure 1).

## RESULTS

### Characterization of Dox-Fu@AuNPs

**Figure 1:**
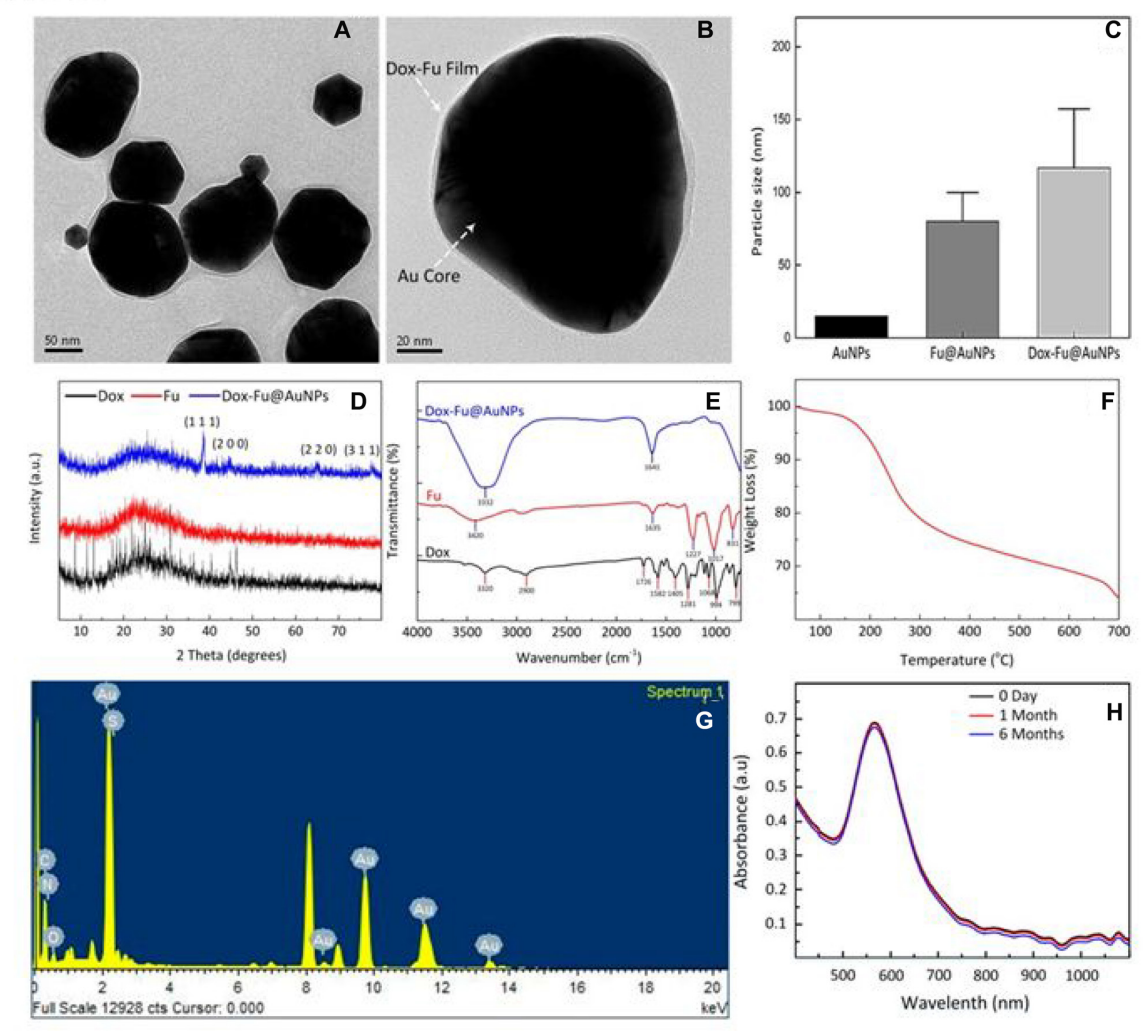
Characterization of synthesized Dox-Fu@AuNPs TEM images of (**A**) Dox-Fu@AuNPs and (**B**) Dox-Fu@AuNPs surrounded with grey shell representing Fu-coated Dox, (**C**) comparison of AuNPs [31], Fu@AuNPs [20], and Dox-Fu@AuNPs estimated by DLS, (**D**) and (**E**) XRD patterns and FT-IR spectra of Dox, Fu, and Dox-Fu@AuNPs, respectively, (**F**) TGA curve of Dox-Fu@AuNPs, (**G**) EDS analysis of Dox-Fu@AuNPs, and (**H**) absorption spectra of Dox-Fu@AuNPs as function of wavelength at various times (0 day, 1 day, and 6 months).

For effective chemo-photothermal therapy of eye tumor, Dox-Fu@AuNPs were characterized in Figure 1. High-resolution TEM (HRTEM) images in Figure 1A demonstrate that Dox-Fu@AuNPs had spherical shape with a size of 101.5 ± 23.2 nm. AuNPs encapsulated by Fu was successfully coated with Dox, which is indicated by white dotted arrows (Figure 1B). The average particle size of Dox-Fu@AuNPs was 116.7 ± 40.6 nm (Figure 1C) whereas the average sizes of both AuNPs and Fu@AuNPs were 15 nm [31] and 82 ± 11.5 nm [20]. Table 1 summarizes the measure particle sizes. Zeta Potential value of Dox-Fu@AuNPs in an aqueous solution was measured to be −46.2 mV, indicating that the surface of AuNPs was mainly coated with negatively charged groups and also responsible for the moderate stability of the nanoparticles in the aqueous solution. XRD patterns of Dox, Fu, and Dox-Fu@AuNPs confirm the crystal structures of the synthesized nanoparticles (Figure 1D). The Dox-Fu@AuNPs sample exhibited the distinctive peaks at 2θ values of 38.6°C, 44.5°C, 64.7°C, and 77.6°C, which corresponded to the (111), (200), (220), and (311) reflection of the crystalline metallic of AuNPs (JCPDS 04-0784). The FT-IR spectra exhibit the characteristic peaks of different functional groups at various positions shown in (Figure 1E). Overall, the spectra of Dox, Fu and Dox-Fu@AuNPs samples displayed strong broadband of O-H stretching of alcohols groups bound to the AuNPs at 3320, 3420, and 3332 cm^–1^, respectively. The peak around 2900 cm^–1^ is attributed to the C-H stretching of alkanes. The band at 1726 cm^–1^ exhibits the stretch of the saturated ester C=O groups. The two peaks at 1641 and 1635 cm^–1^ correspond to the stretching vibration from the primary amines. The bands at 1582 cm^–1^ are assigned to aromatic C-C ring stretching groups. The characteristic peak at 1405 cm^–1^ can be assigned to the bending of –COO^-^ group. The bands at 1227 and 1281 cm^–1^ correspond to the C-H wag alkyl halides. The peaks around 1017 and 1068 cm^–1^ correspond to the C-N stretching of aliphatic amines. The TGA curve shows a significant weight loss of the sample as a function of temperature in Figure 1F. The weight loss was initiated at the temperature of 100°C, which corresponded to the removal of residual moisture in the sample. In addition, the weight showed a slight decrease (about 1.7 %) but induced a sudden weight loss at the temperature between 135 to 300°C due to decomposition of Fu [32]. Then, the gradual weight decrease indicates that the organic compounds from Dox and Fu surrounding Dox-Fu@AuNPs were completely degraded at the higher temperatures. As a result of the temperature increase to 700°C, the percentage of the total weight loss of the coating layer for Dox-Fu@AuNPs was measured to be approximately 35%. Thus, the residual weight of the corresponding AuNPs was 65%. The EDS spectra confirm the presence of Au as the main element signal in Dox-Fu@AuNPs (Figure 1G and Table 2). The fabricated nanoparticles were composed of 88.8 % Au, 9.4 % C, 0.4 % N, 0.2 % O, and 0.3 % S by weight. Absorption spectra of Dox-Fu@AuNPs at various times (0 day, 1 month, and 6 months) demonstrate strong light absorption at 532 nm and absorbance stability regardless of the time (Figure 1H), which means heat generation can be occurred effectively at 532 nm wavelength. Thus, it was confirmed that the fabricated Dox-Fu@AuNPs can be applicable for PTT and PAI. Characterization of the other control particles (i.e., Fu@AuNPs) has been reported in our previous study [20].

**Table 1:** Particle diameter

Sample name	Modification	PD^*^ by TEM	HD^**^ by DLS
Dox	-Fucoidan	101.5 ± 23.2	116.7 ± 40.6
Dox-Fu	-	5.7 ± 1.3	-

^*^PD: particle diameter; ^**^HD: hydrodynamic size of Dox-Fu@AuNPs.

**Table 2:** Atoms analysis of Dox-Fu@AuNPs measured by EDS

Element	EDS	
	Weight (%)	Atomic (%)
Au	88.77	33.57
C	9.36	58.06
N	0.42	2.24
O	1.18	5.50
S	0.27	0.63

### photothermal effects of Dox-Fu@AuNPs

**Figure 2:**
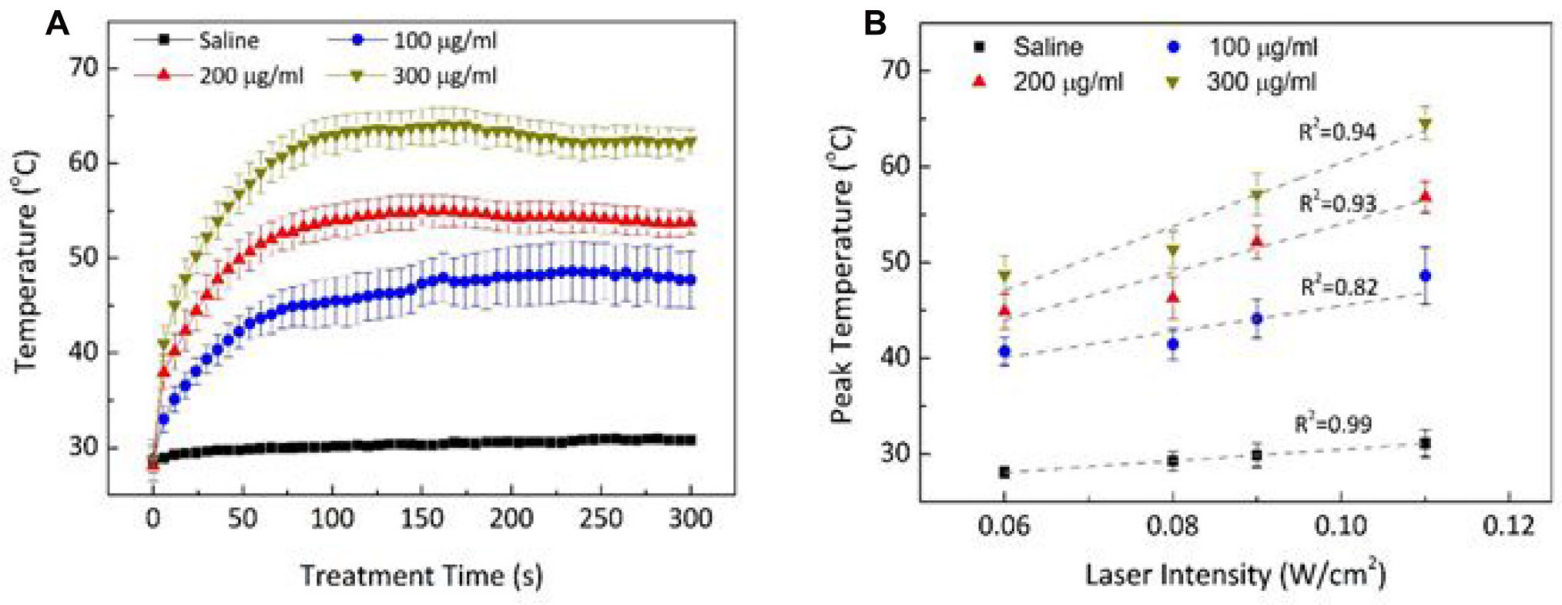
Photothermal effects of Dox-Fu@AuNPs (**A**) Temporal development of Dox-Fu@AuNPs in 1 ml of saline was monitored during laser exposure at 0.11 W/cm^2^ for 5 min. (**B**) The peak temperatures from saline and Dox-Fu@AuNPs in aqueous solution at various concentrations (0 from saline, 100, 200, and 300 µg/ ml) after 5-min laser irradiation was compared as a function of laser intensity.

Thermal responses of Dox-Fu@AuNPs during 532-nm laser irradiation for 5 min were evaluated in terms of temporal development of temperature (Figure 2A) and peak temperature (Figure 2B). The temporal elevation of the temperature during the irradiation [0.11 W/cm^2^; Figure 2A demonstrated that regardless of the concentration, the temperature in the Dox-Fu@AuNPs aqueous solution began to increase rapidly, but after 1 min, the temperature became almost saturated. The corresponding steady-state temperatures were 48.6 ± 3.0, 56.8 ± 1.6, and 64.6 ± 1.7°C for 100, 200, and 300 µg/ml, respectively. Thomsen *et al.* reported that the temperature of approximately 55∼70°C could lead to irreversible damage to tumor cells [33]. The peak temperature almost linearly increased with laser intensity and concentration of Dox-Fu@AuNPs. Control (saline) shows no considerable changes in the temperature during the irradiation. At the lowest irradiance (0.06 W/cm^2^), the temperature increased from 40.7 ± 1.5°C with 100 µg/ml Dox-Fu@AuNPs (thermal gradient = 0.04°C/s) to 48.7 ± 2.0°C with 300 µg/ml Dox-Fu@AuNPs (0.07°C/s). At the highest laser irradiance (0.11 W/cm^2^), the peak temperature significantly increased from 48.6 ± 3.0°C with 100 µg/ml Dox-Fu@AuNPs (0.07°C/s) to 64.6 ± 1.7°C with 300 µg/ml Dox-Fu@AuNPs (0.12°C/s). The maximum temperature increases were estimated to be 16.0, 24.1, and 29.1°C for Dox-Fu@AuNPs samples at 100, 200, and 300 µg/ml, respectively (initial temperature = 28.1°C). Thus, due to the steady-state temperature higher than 65°C, both 200 µg/ml of Dox-Fu@AuNPs and 2-min irradiation (at 0.11 W/cm^2^) could be appropriate conditions to induce the irreversible thermal damage to cancer cells.

### *In vitro* cytotoxicity effect of Dos-Fu@AuNPs

**Figure 3:**
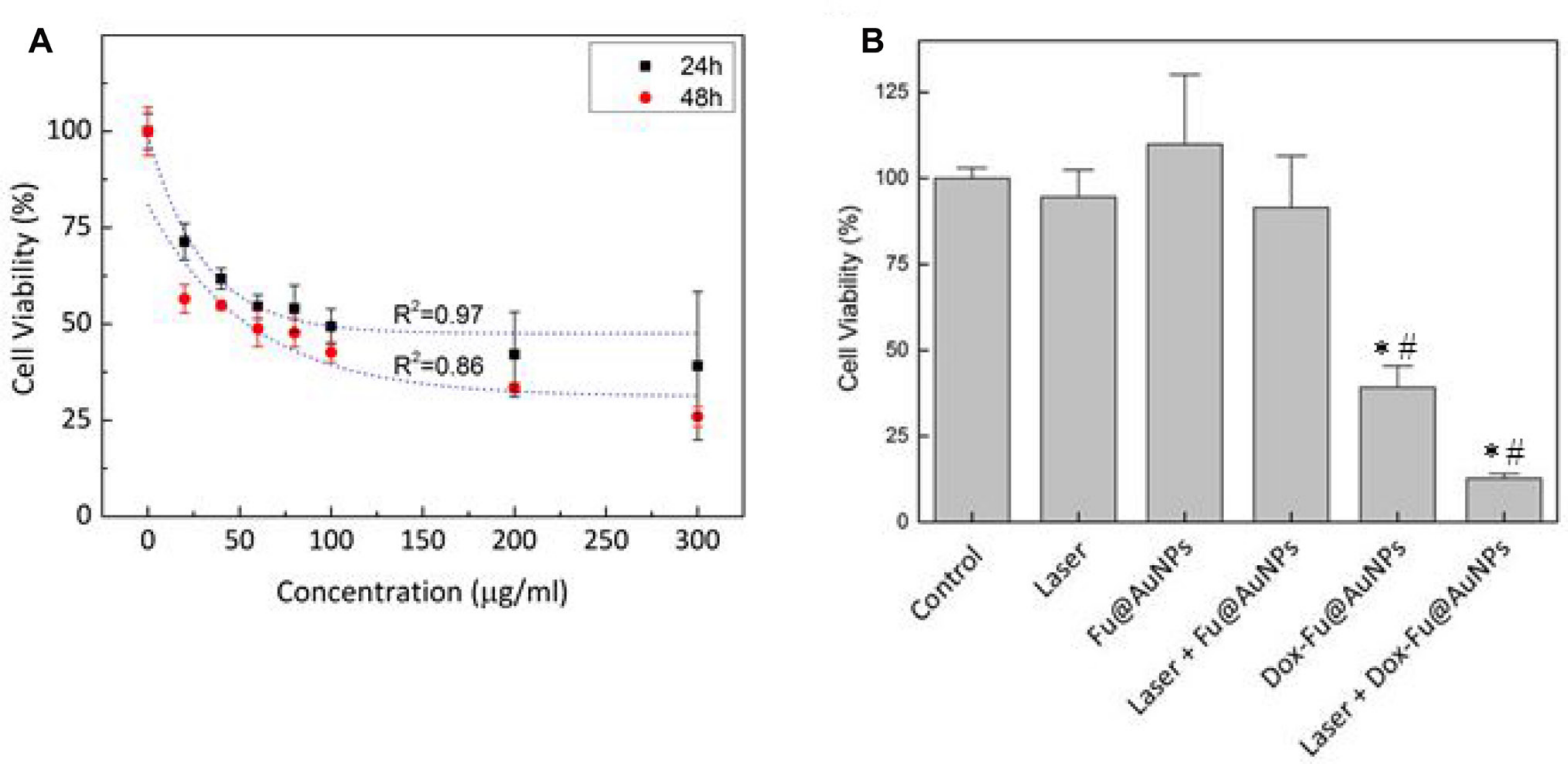
Photothermal and cytotoxicity effects against VX2 cells with Dox-Fu@AuNPs (**A**) The cell viability of Dox-Fu@AuNPs in VX2 at various concentrations (0, 20, 40, 60, 80, 100, 200, and 300 µg/ml) with different incubation times (24 and 48 h) was measured by using MTT assay. (**B**) VX2 cell viability under various treatment conditions (control, laser, Fu@AuNPs, Dox-Fu@AuNPs, Fu@AuNPs-assisted laser irradiation, and 200-µg/ml Dox-Fu@AuNPs-assisted laser irradiation for 2 min) was analyzed by using MTT assay (^*^and ^#^: *p* < 0.05 compared with control and laser group, respectively).

VX2 squamous carcinoma cells and Raw 264.7 cells were tested to investigate anti-tumor effect of Dox-Fu@AuNPs *in vitro* as a function of concentration at two different incubation times (Figure 3A and Supplementary Figure 2). Regardless of incubation time, control (no particles) demonstrated no changes in the cell viability. The viability of the VX2 cells rapidly decreased with the increasing concentrations of Dox-Fu@AuNPs. However, above the concentration of 200 µg/ml, the cell viability became saturated (24 h; cell viability of 100 µg/ml: 49.3 ± 4.6%, 200 µg/ml: 42.1 ± 11%, 300 µg/ml: 39.1 ± 19.2%). In spite of the comparable trend (Figure 3A), the longer incubation time (48 h) further decreased the cell viability by up to 39% (at 300 µg/ml), in comparison with 24 h.

### *In vitro* chemo-photothermal therapy with Dox-Fu@AuNPs

Cell viability was evaluated to assess effect of various conditions on VX2 tumor cells (Figure 3B): control, laser only, Fu@AuNPs only, Dox-Fu@AuNPs only, laser with Fu@AuNPs, and laser with Dox-Fu@AuNPs (concentratio *N =* 200 µg/ml, irradiance = 0.11 W/cm^2^, and irradiation time = 2 min). Both control and laser only exhibited almost insignificant effects (≥90%). In addition, Fu@AuNPs also had no significant thermal damage on the VX2 cells. Fu@AuNPs with laser irradiation hardly promoted cell death possibly due to less significant temperature increase (up to 41°C) in the cells. However, the addition of Dox-Fu@AuNPs noticeably decreased the cell viability. Moreover, Dox-Fu@AuNPs under laser irradiation resulted in the maximal cellular death of 88%, which is up to 7-fold higher than those of the other conditions.

### Hoechst 33342 and PI double staining

**Figure 4:**
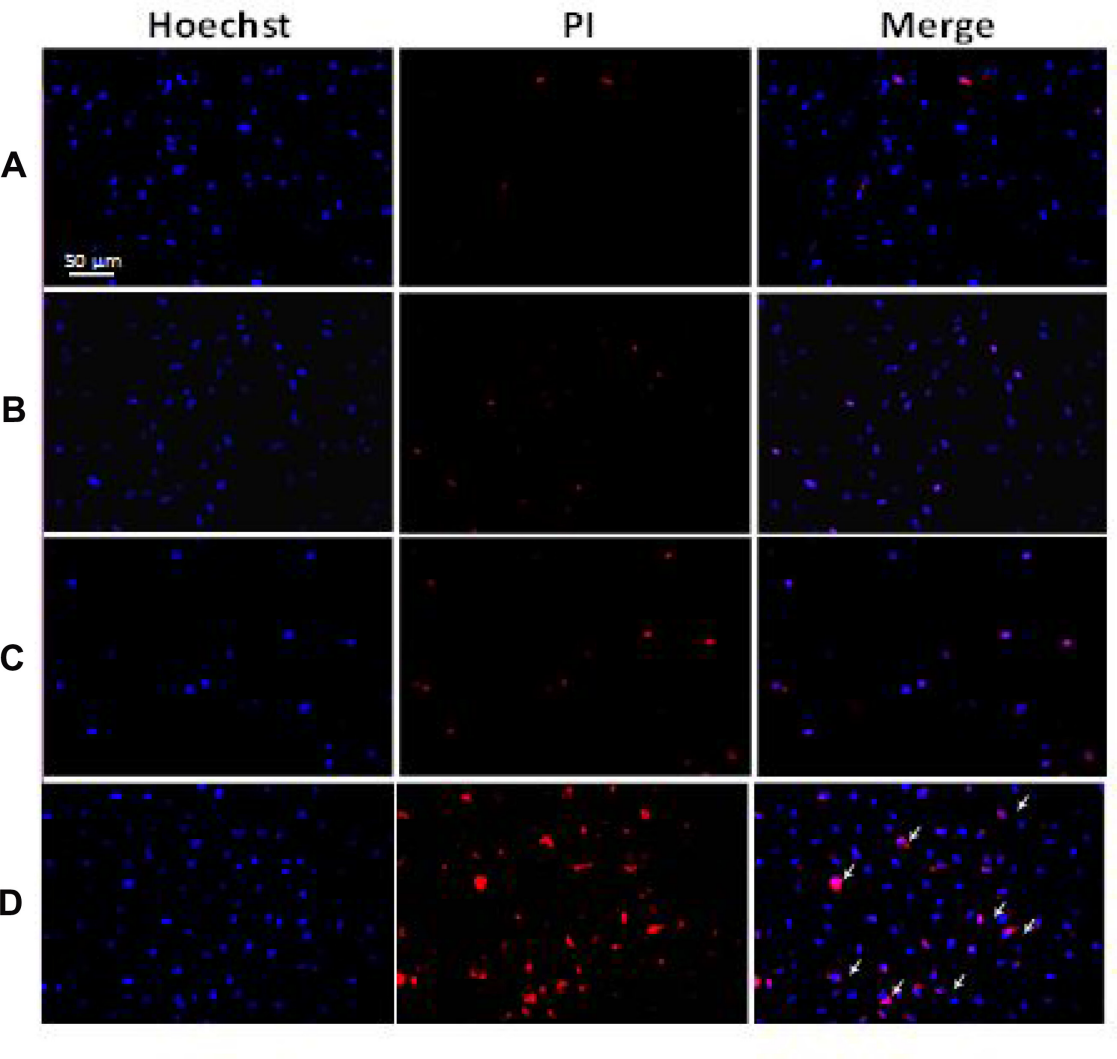
Double staining with Hoechst 33342 and PI on VX2 cells: (**A**) control, (**B**) cells irradiated with laser only (0.11W/cm^2^), (**C**) cells treated with Dox-Fu@AuNPs at 200 µg/ml, and (**D**) cells treated with Dox-Fu@AuNPs at 200 µg/ml followed by laser irradiation (0.11 W/cm^2^ for 2 min). Nuclei were stained with Hoechst 33342 (blue). The dead cells were represented by red staining with PI (20 × ; bar = 50 µm).

The treated VX2 cells were stained with Hoechst 33342 and PI and observed with a fluorescence microscope to evaluate the anti-tumor effect of Dox-Fu@AuNPs-assisted PTT (concentratio *N =* 200 µg/ml, irradiance = 0.11 W/cm^2^, and irradiation time = 2 min) on tumor cells (Figure 4). Blue emission is from the Hoechst dye that stains the nuclei of viable VX2 cells, and red emission from PI staining indicates dead cells. Both control (Figure 4A) and laser only (Figure 4B) displayed clear blue emissions from the nuclei along with minimal red emissions, indicating that cells were viable due to insignificant cytotoxicity. The treated cells with Dox-Fu@AuNPs (no irradiation) exhibited a slight increase in the red fluorescence, thus representing a slight increase in the dead cells (Figure 4C). In contrast, the VX2 cells treated with Dox-Fu@AuNPs and laser irradiation yielded a marked increase in the cellular death. The fluorescence images of the treated VX2 cells were also taken at various positions: the following positions: (a) outside, (b) inside (center), and (c) at the border of irradiation zone (Supplementary Figure 3). The cells located outside the laser beam showed good viability (Supplementary Figure 3A) whereas most of the cells in the area of laser exposure were dead (Supplementary Figure 3B). Supplementary Figure 3C clearly exhibits a mixture of the treated and the non-treated regions at the border, implicating selective antitumor effects as a result of dual-chemo- and photothermal treatments.

### *In vivo* Testing

**Figure 5:**
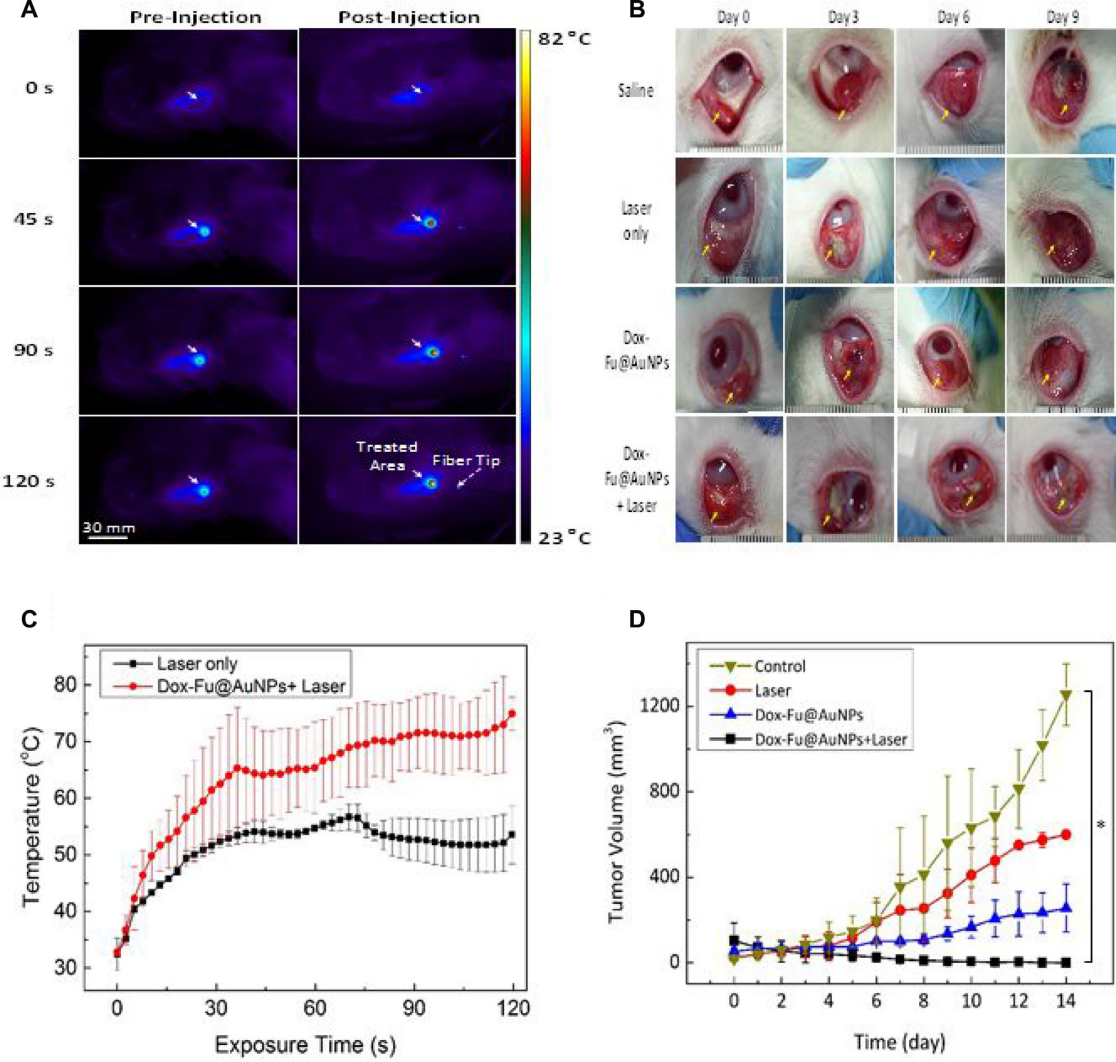
Chemo-photothermal effects of Dox-Fu@AuNPs in rabbit eye tumor model (**A**) *In vivo* infrared thermographic maps of eye tumors before and after injection with Dox-Fu@AuNPs were shown at various times after laser irradiation. (**B**) Representative photographs of the rabbit eye tumors under various conditions (saline as control, laser only, Dox-Fu@AuNPs only, and Dox-Fu@AuNPs with laser irradiation) were shown. (**C**) The graph indicates temporal development of temperature from the irradiated area in the tumor during the laser irradiation (laser only vs. Dox-Fu@AuNPs with laser irradiation: *N =* 20). (**D**) Tumor volume was estimated three times per day over a period of 14 days after each treatment. (^*^p < 0.05 compared with control).

Dual-treatment efficacy of Dox-Fu@AuNPs was assessed with VX2 tumor-bearing rabbits (Figure 5). Figure 5A presents a series of IR thermal images of the tumor region at various times for two groups: laser only (left column) and laser with Dox-Fu@AuNPs (right column). During the laser irradiation, all the groups exhibited a temperature increase in terms of color variations. The thermal lesion had a round shape with a diameter of approximately 10 mm, which was large enough to cover the entire tumor for hyperthermia. Apparently, the laser with Dox-Fu@AuNPs group yielded a 32% higher increase than the laser only group did (i.e., 56.7°C for laser only vs. 75.0°C for laser with Dox-Fu@AuNPs for 2-min irradiation). Temporal elevation of the temperature in the tumors was also monitored in Figure 5C. Overall, both the groups demonstrated that the tumor temperature increased with the irradiation time but became almost saturated around 30 s after the onset of the irradiation. After 2-min irradiation, the laser with Dox-Fu@AuNPs group entailed the temperature of 75.0 ± 2.9°C, which is 30% higher than that of the laser only (56.7 ± 2.3°C; *p* < 0.001). It was noted that the induced temperature due strong light absorption was high enough to cause the irreversible thermal damage to the tumors.

The size of each tumor after various treatments was monitored for 14 days to evaluate the dual-treatment efficacy of Dox-Fu@AuNPs: control (saline), laser only, Dox-Fu@AuNPs only, and laser with Dox-Fu@AuNPs as shown in Figure 5B. Yellow arrows indicate the position of the tumor before and after the treatments. The sizes of the tumor for control, laser only, and Dox-Fu@AuNPs only increased slowly for 6 days after the treatment and then gradually increased. In contrast, the tumor size for laser with Dox-Fu@AuNPs significantly decreased over time (Figure 5B), and the tumor began to disappear at day 9. Tumor volume was also quantified for 14 days to compare therapeutic effects of various conditions (Figure 5B). The tumor sizes slightly increased from approximately 100 mm^3^ to approximately 112 mm^3^ over 5 days and then rapidly increased to approximately 1255, 600, and 256 mm^3^ at day 14 for control, laser only, and Dox-Fu@AuNPs only, respectively. Conversely, the laser with Dox-Fu@AuNPs group demonstrated a significant decrease in tumor growth, and the tumor was completely eradicated within 6 days after the treatment. No tumor recurrence was also observed at day 14.

**Figure 6:**
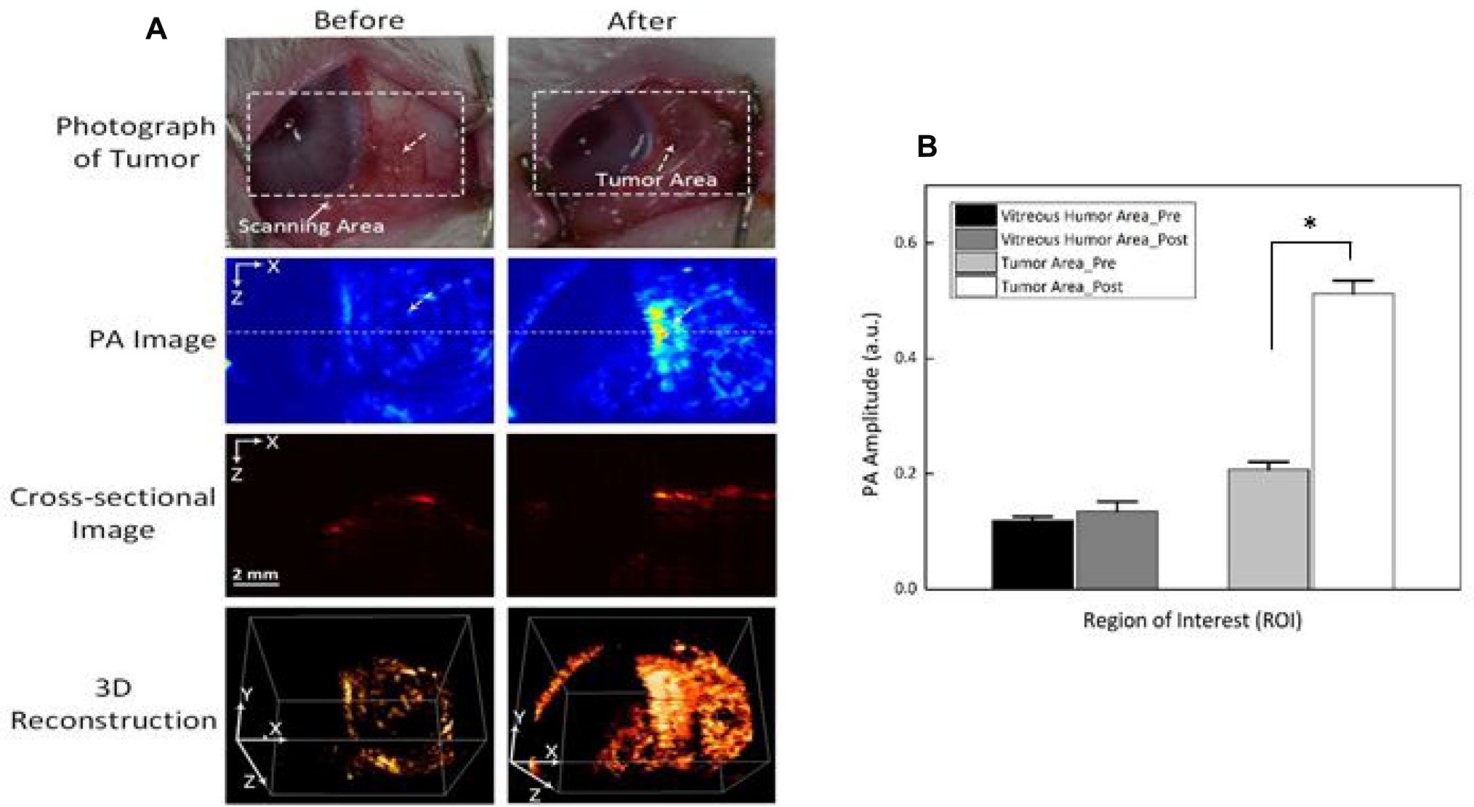
Photoacoustic imaging (PAI) of rabbit eye tumors injected with Dox-Fu@AuNPs (**A**) Photographs of rabbit eye tumor were compared before and after Dox-Fu@AuNPs injection. (**B**) Photoacoustic signals from different positions in the rabbit eye tumor were compared before and after injection of Dox-Fu@AuNPs (^*^p < 0.05 compared with pre-injection).

The feasible application of Dox-Fu@AuNPs as a PA contrast agent was examined in *in vivo* VX2 tumor-bearing rabbit models before and after a single injection of 100 µl Dox-Fu@AuNPs at 200 µg/µl (Figure 6A). White dashed rectangles represent the scanned areas whereas white dotted arrows indicate the position of the tumor. The tumor injected with Dox-Fu@AuNPs created higher PA signals, compared with the pre-injection that showed an unclear image due to the lack of intrinsic chromophores at 532 nm. The position of the tumor after the injection was ostensibly visualized with high contrast due to strong light absorption from both the blood and the Dox-Fu@AuNPs. Cross-sectional images demonstrate the corresponding PA B-scan images acquired from the dotted lines to provide the axial penetration depth of the injected Dox-Fu@AuNPs. The post-injection PA image clearly shows the tumor filled with Dox-Fu@AuNPs whereas the cross-sectional image of the tumor before the injection is blurred and difficult to differentiate. The PA image of the tumor after the injection provides more than a 2-fold deeper image depth in the tissue, compared with the pre-injection (i.e., imaging depth = 2.8 ± 0.1 mm for post-injection vs. 1.4 ± 0.1 mm for pre-injection; *p* < 0.001). A 3D rendering PA image was reconstructed from a sequence of the B-scan cross-sectional images. The 3D image reconstruction was used to visualize the margins of the tumor and to evaluate the spatial distribution of the nanoparticles in the tumor. Obviously, the entire structure of the tumor volume was clearly visible upon the injection of Dox-Fu@AuNPs. In addition, the image after injection seemed larger, implicating the effective diffusion of Dox-Fu@AuNPs upon the injection. Quantitative measurements of the PA signals from regions of interest (ROIs) in Figure 6B were performed to differentiate the tumor areas from the surrounding vitreous humor (Figure 6B). The PA image contrast of the post-injected tumor was approximately 2.6 times higher than that of the surrounding vitreous humor (i.e., PA amplitudes = 0.51 ± 0.02 for post-injection vs. 0.14 ± 0.02 for vitreous humor, *p* < 0.001). In contrast, the PA contrast of the pre-injection slightly increased, in comparison with that from of the surrounding area (i.e., PA amplitudes = 0.21 ± 0.01 for pre-injection vs. 0.12 ± 0.01 for vitreous humor; *p* < 0.001).

Histological analysis on the treated tumors at day 14 was also performed to investigate antitumor effects of Dox-Fu@AuNPs (Supplementary Figure 4). Control images showed no evidence of anatomic disorganization of the tumor tissues. It was clearly observed that the morphology of cellular structures was unchanged and most nuclei were easily found, indicating that cancer cells were barely affected in the absence of Dox-Fu@AuNPs. In contrast, the samples treated with Dox-Fu@AuNPs only presented minute necrotic foci. The tissues treated with laser only and a combination of Dox-Fu@AuNPs and laser irradiation demonstrated distinct necrosis (Dox and thermal effects) in a superficial portion of the tumor and no or minimal damage to the peripheral tissues. The depth of the necrosis was estimated to be approximately 0.4 mm and 1.5 mm for the tissues treated with laser-only and with laser with Dox-Fu@AuNPs, respectively. Thus, the tissue necrosis induced by the combined treatment was approximately 4-fold thicker than that with laser only.

## DISCUSSION

AuNPs have been investigated as drug carriers, photosensitizer agents, and contrast agents for tumor targeting, imaging, and selective therapy [16]. As strong absorption of visible light by AuNPs can lead to heat generation, the unique plasmonic effect can be applied for PTT and PAI on solid tumors [29, 34]. The current study developed and synthesized AuNPs coated with Dox-loaded Fu for enhancing the synergistic effects of chemotherapy, the augmented heating for PTT, and the enhanced noninvasive photoacoustic image contrast for tumor margin identification and dual-treatment *in vivo*.

Recently, a number of inorganic nanoparticles have been widely investigated in imaging and treatment of tumors both *ex vivo* and *in vivo*, including gold nanoparticles (spheres, shells, rods, and cages) [35], carbon-based materials (SWNT, graphene, and carbon dot) [36, 37], magnetic nanoparticles [38], quantum dots, and ceramic nanoparticles [39]. However, the current nanoparticles are still limited by long-term toxicity, limited imaging resolution and penetration depth, and fast clearance by phagocytes (SPIO) [40]. Overcoming these challenges requires the development of an essential agent that reduces cytotoxicity, produces sufficient photoacoustic signals, and enhances photothermal efficiency. A great number of studies have reported the conjugation of AuNPs with different materials such as Prussian blue [29], polyelectrolyte [41], or thiolate chitosan as an alternative approach to reduce cytotoxicity. Other studies have focused on enhancing the biocompatibility and biodegradability of synthesized agents by loading them with organic dyes such in the case of heparin-folic acid-IR-780 nanoparticles [42] or by loading hollow silica nanoparticles with hydrophobic phthalocyanine (Pc@HSNs) [43] or Zn(II)-phthalocyanine [44] or coating them with polyethylene glycol (PEG), including PEGylated WS2 nanosheets [45]. Most of the available photosensitizers are hydrophobic in nature and easily aggregate in physiological saline. To overcome these limitations, biocompatible AuNPs were synthesized by using a natural Fu as a conjugating and stable carrier for effective PTT therapy and PAI. Fu is a natural polymer with antitumor activity, no toxicity, and excellent biocompatibility and stability for cancer treatment [46–48]. Therefore, Fu plays a key role as a surface coating and reducing agent for the AuNPs core formation and leads to reduction of the toxicity of AuNPs and to the enhanced anti-tumor effects. In addition, to achieve effective tumor removal, the anti-cancer agent, Dox was integrated into Fu-coated AuNPs with over 90% loading efficacy, which was validated by a previous study [20]. The current study demonstrated a comparable cytotoxic effect of Dox over 200 µg/ml (Figure 3A). In fact, the saturation concentration of Dox for conjugation within nucleus was reported to be 340 µM (i.e., 184.8 µg/ml) [49]. If the loading efficacy of Dox on Fu@AuNPs is considered over 90% [20], the cytotoxic effect could become comparable over 200 µg/ml of Dox-Fu@AuNPs (i.e., 180 µg/ml of Dox).

PTT on Dox-Fu@AuNPs caused cell death by damaging intracellular biomolecules and disturbing the membranes of intracellular organelles. The cell death was characterized as cell shrinkage, nuclear pyknosis (chromatin condensation), karyorrhexis (nuclear fragmentation), nuclei cleavage, and autophagic and nonlysosomal disintegration by microscopic observations (Figure 4). Markovic *et al.* reported that the mechanism of photothermal killing of cancer cells involved oxidative stress and mitochondrial membrane depolarization, resulting in apoptotic and necrotic cell death characterized by caspase activation, DNA fragmentation, and cell membrane damage [50]. Thus, PTT increased apoptosis and necrosis through mitochondrial membrane depolarization. The loss of mitochondrial membrane potential, DNA fragmentation, and phosphatidylserine exposure were observed in the PTT-treated cells, which led to morphological and biochemical changes in the dying cells. However, the proposed particles were still associated with a relatively wide distribution in size. Thus, the controlled conjugation of Dox to Fu@AuNPs is currently under investigation to attain a uniform size of Dox-Fu@AuNPs. Although various studies have conducted PI fluorescence staining for detection of cellular death and cell cycle [20, 51, 52], the fluorescent emissions between PI and Dox are still undistinguishable. Therefore, flow cytometry or western blot will further be performed to define the cellular death and to confirm the current findings.

During 532-nm irradiation, Dox-Fu@AuNPs yielded no considerable heating effect at 0.06 W/cm^2^, irrespective of concentration. However, the photothermal response was greatly enhanced as both irradiance and concentration were increased to 0.11 W/cm^2^ and 200 µg/ml, respectively (Figure 2A). Thus, small (∼101 nm) Dox-Fu@AuNPs responded well to laser irradiation, and the temperature increase varied in an irradiance- and concentration-dependent manner. This result confirmed that only the highest concentration of 300 µg/ml reached tumor denaturation temperature. Yuan *et al.* showed that a laser irradiance of approximately 433 W/cm^2^ was required to reach the threshold temperature for tumor tissue denaturation (>55°C) [53]. However, the application of high intensity laser light for cancer treatment is always associated with thermal injury to the surrounding healthy tissues. In contrast, the proposed method used a relatively lower light intensity to photothermally treat small-sized eye tumors and to minimize the thermal injury to the peripheral tissues. To reach the threshold temperature for the tumor denaturation, the laser only treatment would require the irradiance of 0.55 W/cm^2^, based upon the linear regression line [i.e., *y* = 24.5 + 60.1 × *x*, where *x* = laser irradiance and *y* = peak temperature; Figure 2B]. On the other hand, the application of Dox-Fu@AuNPs was able to reduce the irradiance down to 0.11 W/cm^2^ (i.e., 4.5 times lower) due to efficient optical energy coupling. Therefore, the proposed nanoparticles could be the potential photothermal agent to treat the eye tumors with minimal thermal injury to the adjacent tissue.

*In vivo* experiments presented that the combination of the laser irradiation and the nanoparticles induced temperature elevation up to 42.2°C in less than 1 min, which is approximately 1.7 times high as that with laser only. The temperature rise can be attributed to the concurrent absorption of laser light by hemoglobin in the tumor and by Dox-Fu@AuNPs, leading to reduction of the treatment time, whereas no temperature increase was observed from the cells under laser irradiation without nanoparticles (laser only) due to weak absorption of the clear cells [54–56]. Unlike other conditions, the laser with Dox-Fu@AuNPs removed the tumors within six days without noticeable toxicity to the animals and recurrence over 14 days. This finding confirmed that the both chemo- and photothermal effects successfully inhibited tumor growth without any adverse effects. It was also observed that Dox-Fu@AuNPs with or without laser irradiation was much greater than that of laser only due to anti-tumor effect of Dox. Importantly, all the treatments that used a single dose of the nanoparticles demonstrated a nonspecific effect to the surrounding tissues [42]. It is worth mentioning that chemotherapy-combined PTT can significantly improve treatment efficacy on eye tumors [57]. In addition, the current histological analysis merely presented the resultant necrosis 14 days after the treatment. Thus, TUNEL assay will further be conducted to detect apoptosis after the combined treatment. Furthermore, the acquired PA images (Figure 6) demonstrated that Dox-Fu@AuNPs obviously increased the image contrast in the tumor regions by 2.5 fold. The augmented contrast could result from strong absorption of 532 nm light by both blood and Dox-Fu@AuNPs in the tumor, which corresponds to the peak absorption wavelength of hemoglobin and Dox-Fu@AuNPs, as shown in Figure 1H and also reported by Prahl *et al.* [58]. This result implies that Dox-Fu@AuNPs could serve as effective contrast agents to enhance non-invasive PA imaging for cancer diagnosis. However, more effort is still required to systematically examine the potential long-term toxicity of the synthesized nanoparticles and the feasibility of the dual-theranostic agent at various doses in the animal models. In addition, the current histological analysis merely presented the resultant necrosis 14 days after the treatment. Thus, TUNEL assay will further be conducted to detect apoptosis after the combined treatment. Since solid tumors have wider and looser blood vessels (i.e., leaky vasculature; 200 nm ∼ 1.2 µm in size depending on tumor type) than normal tissue does (<10 nm in size) [59, 60], the current nanoparticles (116.7 ± 40.6 nm) may readily be transferred to and accumulated into the tumor due to enhanced permeability and retention (EPR) effect [61]. Intraperitoneal or intravenous injection of Dox-Fu@AuNPs will thereby be performed to identify the feasible detection of the tumor margin with PAI. Furthermore, synthesis of antibody with Dox-Fu@AuNPs will be conducted to enhance selective tumor targeting for the effective dual-treatment [62].

## CONCLUSIONS

The current study investigated the feasible application of Dox-Fu@AuNPs for dual-treatment (chemo- and photothermal-) on eye tumor *in vitro* and *in vivo*. The fabricated Dox-Fu@AuNPs presented good stability in physiological environments and were non-cytotoxic to normal cells at the tested concentrations. On account of strong laser light absorption and high thermal conversion efficiency even at low doses, Dox-Fu@AuNPs generated both chemo and photothermal effects to completely remove the tumors. Both high photostability and molecular extinction coefficient enabled Dox-Fu@AuNPs to augment photoacoustic image contrast for the feasible identification of the tumor margins for the treatment. Therefore, Dox-Fu@AuNPs can be effective and safe nano-theranostic agents for imaging-guided dual-treatments for eye cancer.

## MATERIALS AND METHODS

### Chemical materials

Fu extracted from *Fucus vesiculosus* was obtained from Sigma (Sigma, St. Louis, Mo, U.S.A.). Chloroauric acid trihydrate (HAuCl_4_∙3H_2_O, > 99.9%) and doxorubicin hydrochloride (Dox·HCl) were purchased from Sigma-Aldrich Co. (St. Louis, MO, USA). Dulbecco’s modified eagle’s medium/F12 (DMEM-F12) was obtained from Cellgro (Mediatech, Massachusetts, USA). Dulbecco’s modified eagle’s medium (DMEM), trypsin–ethylenediaminetetraacetic acid (trypsin-EDTA), antibiotics, fetal bovine serum (FBS), and phosphate-buffered saline (PBS) were purchased from Gibco BRL, Life Technologies (Grand Island, NY, USA). 3-(4,5-Dimethyl-2-thiazolyl)-2,5-diphenyl-2H-tetrazolium bromide (MTT), dimethyl sulfoxide (DMSO), Hoechst 33342, and propidium iodide (PI) were obtained from Sigma-Aldrich. Double-distilled water was used for all aqueous solutions in the experiments. All chemicals were used directly as received without further purification.

### Synthesis of Dox-Fu@AuNPs

0.005 g of Fu was poured into 10 ml HAuCl_4_∙3H_2_O aqueous solution at a concentration of 1 × 10^–4^ M, and the solution was stirred at 80°C for 30 min on a magnetic hot plate. The synthesized AuNPs were isolated by centrifuging the mixed solution at 13,000 rpm for 30 min. Then, several cycles of washing with deionized water and centrifuging were performed to remove excess fucoidan and unreacted particles for the further experiments. Filtration was performed for 24 h through a dialysis tube with a 12,000 Da molecular weight cutoff to remove ionic impurities. Dox-Fu@AuNPs were prepared by adding Dox·HCl to the solution containing the fucoidan-coated AuNPs. The final concentration of Dox was adjusted to 10^−4^ M in a solution. The resulting mixture was stirred for 2 min to obtain a homogeneous distribution of AuNPs and then incubated for 24 h at room temperature. The resulting solution was centrifuged at 10,000 rpm for 15 min to remove excess non-reacted compounds. The acquired pellet after centrifugation was distinguished from the supernatant solution and re-dispersed in deionized water prior to the further characterization [22].

### Characterization of Dox-Fu@AuNPs

To select the excitation wavelength for PTT, the absorbance of Dox-Fu@AuNPs was measured from 300 to 1100 nm by using a spectrometer (XS2, BioTek, Winooski, VT, USA). High-resolution transmission electron microscopy (HR-TEM) (JEM 2010, JEOL Ltd, Tokyo, Japan) was used to visualize the fabricated nanoparticles. The HR-TEM images provided the estimated size and morphology of the nanoparticles. The median size of the nanoparticles was then calculated with Image J (National Institute of the Health, Bethesda, MD, USA). For comparison, the particle size was analyzed again with dynamic light scattering (DLS) by using an electrophoretic light scattering spectrophotometer (ELS-8000, OTSUKA Electronics Co. Ltd., Japan) at a fixed angle of 90°C and room temperature. Zeta potential (ZP) values of Dox-Fu@AuNPs were measured by utilizing an electrophoretic light scattering spectrophotometer (ELS-8000, OTSUKA Electronics Co. Ltd., Japan).

The elemental analysis of the synthesized Dox-Fu@AuNPs was evaluated by using energy dispersive x-ray spectroscopy (EDS; Hitachi, S-2400, Japan/Kevex Ltd, Sigma). The surface elemental compositions and impurities of Dox-Fu@AuNPs were identified by using EDS analysis in a specific scan area on the SEM sample at 20 keV. The crystalline structure of the powdered nanoparticles was analyzed by using an X-ray power diffraction (XRD) machine (X’Pert-MPD System, Philips, Almelo, Netherlands). The chemical functional groups of the nanoparticles were determined by using Fourier-transform infrared spectroscopy (FT-IR) spectrometer (FT/IR 6100, Jasco, Easton, Pennsylvania, USA) in a diffuse reflectance mode. The contents of the nanoparticles (i.e., organic and inorganic elements) were characterized by performing thermogravimetric analysis (TGA) (TGA7, Pyris 1, Perkin Elmer, Waltham, Massachusetts, USA). The TGA analysis of the nanoparticles was determined by the nanoparticles placed in an alumina pan under the nitrogen atmosphere and heated from room temperature to 700°C at a ramping time of 10°C/min.

### Light source

For laser irradiation, a quasi-cw Q-switched 532-nm laser system (GreenLight PV^®^, American Medical Systems, Inc., San Jose, USA) was employed at various irradiances (i.e., 0.06, 0.08, 0.09, and 0.11 W/cm^2^) and times (i.e., 2, 3, and 5 min). A multimode 600-µm optical fiber (NA = 0.22) was used to deliver the laser light into each sample. The spot size was estimated to be 10.2 mm^2^. To assess photothermal effects of the synthesized Dox-Fu@AuNPs, four different concentrations of Dox-Fu@AuNPs in solution [0 (saline), 100, 200, and 300 µg/ml] were initially tested with the laser irradiation. The samples were poured in the wells of a sterile 96-well plate (total volume of 100 µl per each well). An infrared thermal camera (FLIR A300, FLIR System, Inc., Sweden) was used to monitor spatio-temporal development of temperature in the aqueous solution during the laser exposure.

### *In vitro* chemo-photothermal therapy

To measure cell viability of Dox-Fu@AuNPs without laser irradiation, VX2 cells and Raw 264.7 cells were seeded into a 96-well culture plate at a density of 2 × 10^4^ cells/well and 5 × 10^4^ cells/well in a 100 µl of culture medium, respectively. The seeded cells were then washed with PBS and incubated with the Dox-Fu@AuNP solution at various concentrations (0, 20, 40, 60, 80, 100, 200, 300 µg/ml) for 24 or 48 h at 37°C in the humidified atmosphere of 5% CO_2_. The cells without Dox-Fu@AuNPs were used as a control. After various incubation times, MTT tetrazolium bromide solution (1 mg/ml) was added to each well. The cells were incubated for 4 h, and DMSO was replaced to dissolve formazan crystal. The optical density was quantified at 570 nm by using an ELISA micro plate reader (SpectraMax, 340, Molecular Device, Sunnyvale, CA, USA). The relative cell viability was calculated and compared with that of a non-treated blank group. To evaluate chemo-photothermal therapy on the cells, VX2 cells were seeded in to 96-well culture plate at a density of 2 × 10^4^ cells/well. The seeded cells were incubated with a 200 µg/ ml concentration of Dox-Fu@AuNPs for 4 h at 37°C. The treated cells were then washed with PBS and fresh media was replaced. The cultured cells were then placed in a water bath maintained at 37°C prior to laser treatment. Laser light at an irradiance of 0.11 W/cm^2^ was used to illuminate the samples for 2 min. To assess cell death, MTT assay was performed after 24 h incubation.

### Hoechst 33342 and PI staining

VX2 cells were cultured in 6-well plates at 2 × 10^5^ cells/well and incubated for 24 h at 37°C in the humidified atmosphere of 5% CO_2_. The cells were then treated with Dox-Fu@AuNP solution at the final concentration of 200 µg/ml and further incubated for 4 h. The cultured cell plates were washed three times with PBS to remove unattached nanoparticles before double-staining with Hoechst 33342 and PI fluorescent dyes. After washing, 300 µl of 10 µg/ml Hoechst solution was added to the cells and incubated for another 20 min at 37°C in the dark environment. The cells were then washed twice with PBS, and 300 µl PI (10 µg/ml) was added. The sample was incubated for an additional 10 min at 37°C. Finally, the stained cells were washed three times and observed with a Leica fluorescence microscope equipped with a DFC450C color digital camera (Leica, Wetzlar, Germany).

### *In vivo* testing

24 New Zealand White rabbits (3-4 months old and 2.2-2.6 kg) were used for *in vivo* PTT testing and purchased from Taesung Laboratory Animal Science (Busan, Korea). VX2 tumor cells were used for tumor inoculation into eyes of each animal. The cultured VX2 cells were collected by using a centrifuge at 750 rpm for 5 min. A suspension was prepared of 1.0 × 10^7^ tumor cells in 500 µl HBSS (Hank’s balanced salt solution, Gibco^®^ Thermo Fisher Scientific). To create tumors in the eyes of the rabbits, each rabbit was intramuscularly anesthetized with 10 mg/kg ketamine and 3 mg/kg xylazine and secured in a supine position, and their conjunctival areas were exposed under visual guidance. Using a 27-gauge needle, 0.4 ml of a suspension of VX2 cells (1 × 10^7^) was injected into the subconjunctival space directly over the pars plana. When the tumor grew to a volume of approximately 100 mm^3^, the tumor-bearing rabbit eyes were randomly divided into the following four experimental groups: (1) control group injected with saline and without laser application (*N =* 5), (2) treatment with laser only (2 min; *N =* 6), (3) intratumoral injection of 100 µl Dox-Fu@AuNPs dispersed in saline at 200 µg/ml (*N =* 5) without laser irradiation, and (4) injection of Dox-Fu@AuNPs (100 µl, 200 µg/ml) and laser irradiation (2 min; *N =* 8). All rabbit studies satisfied the guidelines of the Korean National Institutes of Health (KNIH) on the care and use of laboratory animals. The experimental procedures were approved by the Committee on Animal Research of the College of Medicine at Kosin University (Permit number: KMAP–16-24).

For *in vivo* testing, all the rabbits were anesthetized with an intramuscular injection of ketamine (35 mg/kg) and xylazine (5 mg/kg) prior to Dox-Fu@AuNP intratumoral injection. Twenty minutes after the injection, the rabbit models with and without the injected nanoparticles were illuminated with a 532-nm laser system at 0.11 W/cm^2^ (irradiation time of 2 min and beam size of 10.2 mm^2^). An IR thermal camera was employed to real-time monitor temperature development of the tissue during the laser irradiation. Only a single treatment was given to all the animals. The growth of tumors was examined by a caliper every day for 14 days after the treatments. The tumor volume was calculated by using the formula of *v* = (*l* × *w*^2^)/2, where *v, l,* and *w* represent the tumor volume (mm^3^), tumor length (mm), and tumor width (mm), respectively [17].

To identify tumor margins before treatment, a custom-made photoacoustic imaging (PAI) system was used on the tumor-bearing eyes *in vivo*. A detailed description of the system was reported in previous study [63]. To maintain anesthesia during the *in vivo* experiments, anesthetic (i.e., ketamine = 17.5 mg/kg/h and xylazine = 2.5 mg/kg/h) was injected at an interval of 30 min to the animals. Prior to the experiments, the temperature of each rabbit was maintained by using an electric heating pad. After the anesthesia, the rabbits were positioned on the sample stage, and the areas of interest were fixed with transparent tape to stabilize and to minimize any breathing or other motion artifacts. A water bath with an open bottom wrapped with plastic film was placed on the top of the eye tumor. The ultrasound gel was sandwiched between the water bath and the tumor region. An ultrasound transducer was mounted in the water bath, allowing it to move freely in 3D while not applying any physical pressure on the rabbit. Then, the targeted regions were photoacoustically imaged. After acquiring the control PA image, Dox-Fu@AuNPs (0.1 ml and 200 µg/ml) were intratumorally injected into the tumors with a syringe. Twenty minutes after the injection, PAI of the injected tumor was performed. After the *in vivo* experiments, each rabbit was immediately returned to its kennel. To distinguish the position between the normal tissue and the tumor tissue, image segmentation was performed as post-image processing using Matlab software (MathWorks, Massachusetts, USA)

### Histological analysis

To evaluate *in vivo* treatment efficacy of Dox-Fu@AuNPs, all rabbits with eye tumors were euthanized with overdose with CO_2_ gas 14 days after the testing. The eye tumor tissues were removed aseptically for histological examination. The acquired samples were fixed in 10% neutral buffered formalin (VWR, Radnor, PA) for two days. The fixed tissues were cross-sectionally cut in 3 mm sections and embedded in paraffin. Then, the paraffin-embedded tissues were sliced to a thickness of 4 µm and stained with hematoxylin and eosin (H&E). Each slide was evaluated by using an Olympus BX51 light microscope (Olympus Corp., Tokyo, Japan).

### Statistical analysis

All experiments were executed and repeated three times, and data were expressed as mean ± standard deviation (SD). For non-parametric statistical analysis, Mann-Whitney *U* test in conjunction with SPSS (Ver 22, IBM Corporation, Armonk, New York) was used, and *p* < 0.05 was considered statistically significant.

## SUPPLEMENTARY MATERIALS FIGURES



## References

[1] Triozzi PL, Eng C, Singh AD (2008). Targeted therapy for uveal melanoma. Cancer Treat Rev.

[2] Krag DN, Meijer SJ, Weaver DL, Loggie BW, Harlow SP, Tanabe KK, Laughlin EH, Alex JC (1995). Minimal-access surgery for staging of malignant melanoma. Arch Surg.

[3] Emery AF, Kramar P, Guy A, Lin J (1975). Microwave induced temperature rises in rabbit eyes in cataract research. J Heat Transfer.

[4] Finger PT (1997). Radiation therapy for choroidal melanoma. Surv Ophthalmol.

[5] Lagendijk J (1982). A mathematical model to calculate temperature distributions in human and rabbit eyes during hyperthermic treatment. Phys Med Biol.

[6] Wilson MW, Hungerford JL (1999). Comparison of episcleral plaque and proton beam radiation therapy for the treatment of choroidal melanoma. Ophthalmology.

[7] Scott JA (1988). The computation of temperature rises in the human eye induced by infrared radiation. Phys Med Biol.

[8] Nie S, Xing Y, Kim GJ, Simons JW (2007). Nanotechnology applications in cancer. Annu Rev Biomed Eng.

[9] Huang X, El-Sayed IH, Qian W, El-Sayed MA (2006). Cancer cell imaging and photothermal therapy in the near-infrared region by using gold nanorods. J Am Chem Soc.

[10] O’Neal DP, Hirsch LR, Halas NJ, Payne JD, West JL (2004). Photo-thermal tumor ablation in mice using near infrared-absorbing nanoparticles. Cancer Lett.

[11] Kim JW, Galanzha EI, Shashkov EV, Moon HM, Zharov VP (2009). Golden carbon nanotubes as multimodal photoacoustic and photothermal high-contrast molecular agents. Nat Nanotechnol.

[12] Li Y, Lu W, Huang Q, Li C, Chen W (2010). Copper sulfide nanoparticles for photothermal ablation of tumor cells. Nanomedicine.

[13] Yang K, Zhang S, Zhang G, Sun X, Lee ST, Liu Z (2010). Graphene in mice: ultrahigh *in vivo* tumor uptake and efficient photothermal therapy. Nano Lett.

[14] Lakshmanan SB, Zou X, Hossu M, Ma L, Yang C, Chen W (2012). Local field enhanced Au/CuS nanocomposites as efficient photothermal transducer agents for cancer treatment. J Biomed Nanotechnol.

[15] Li L, Rashidi LH, Yao M, Ma L, Chen L, Zhang J, Zhang Y, Chen W (2017). CuS nanoagents for photodynamic and photothermal therapies: Phenomena and possible mechanisms. Photodiagnosis Photodyn Ther.

[16] Boisselier E, Astruc D (2009). Gold nanoparticles in nanomedicine: preparations, imaging, diagnostics, therapies and toxicity. Chem Soc Rev.

[17] Jing L, Liang X, Deng Z, Feng S, Li X, Huang M, Li C, Dai Z (2014). Prussian blue coated gold nanoparticles for simultaneous photoacoustic/CT bimodal imaging and photothermal ablation of cancer. Biomaterials.

[18] Hwang S, Nam J, Jung S, Song J, Doh H, Kim S (2014). Gold nanoparticle-mediated photothermal therapy: current status and future perspective. Nanomedicine.

[19] Atashrazm F, Lowenthal RM, Woods GM, Holloway AF, Dickinson JL (2015). Fucoidan and cancer: a multifunctional molecule with anti-tumor potential. Mar Drugs.

[20] Li B, Lu F, Wei X, Zhao R (2008). Fucoidan: structure and bioactivity. Molecules.

[21] Lu KY, Li R, Hsu CH, Lin CW, Chou SC, Tsai ML, Mi FL (2017). Development of a new type of multifunctional fucoidan-based nanoparticles for anticancer drug delivery. Carbohydr Polym.

[22] Manivasagan P, Bharathiraja S, Bui NQ, Jang B, Oh YO, Lim IG, Oh J (2016). Doxorubicin-loaded fucoidan capped gold nanoparticles for drug delivery and photoacoustic imaging. Int J Biol Macromol.

[23] Tengdelius M, Gurav D, Konradsson P, Påhlsson P, Griffith M, Oommen OP (2015). Synthesis and anticancer properties of fucoidan-mimetic glycopolymer coated gold nanoparticles. Chem Commun.

[24] Zou L, Wang H, He B, Zeng L, Tan T, Cao H, He X, Zhang Z, Guo S, Li Y (2016). Current approaches of photothermal therapy in treating cancer metastasis with nanotherapeutics. Theranostics.

[25] Upadhyay KK, Bhatt AN, Mishra AK, Dwarakanath BS, Jain S, Schatz C, Le Meins JF, Farooque A, Chandraiah G, Jain AK (2010). The intracellular drug delivery and anti tumor activity of doxorubicin loaded poly (γ-benzyl l-glutamate)-b-hyaluronan polymersomes. Biomaterials.

[26] Tian Y, Li S, Song J, Ji T, Zhu M, Anderson GJ, Wei J, Nie G (2014). A doxorubicin delivery platform using engineered natural membrane vesicle exosomes for targeted tumor therapy. Biomaterials.

[27] Duncan R (2006). Polymer conjugates as anticancer nanomedicines. Nat Rev Cancer.

[28] Minchinton AI, Tannock IF (2006). Drug penetration in solid tumours. Nat Rev Cancer.

[29] Pasut G, Veronese F (2007). Polymer–drug conjugation, recent achievements and general strategies. Prog Polym Sci.

[30] Kang SJ, Grossniklaus HE (2010). Rabbit model of retinoblastoma. BioMed Research International.

[31] Sokolov O, Selivanov N, Bogatyrev V, Selivanova O, Velikorodnaya Y, Pocheptsov A, Filatov B, Shchyogolev S, Dykman L (2016). Synthesis and study on activity *in vitro* of the high purity human butyrylcholinesterase conjugated with gold nanoparticles. Dokl Biochem Biophys.

[32] Zayed A, Muffler K, Hahn T, Rupp S, Finkelmeier D, Burger-Kentischer A, Ulber R (2016). Physicochemical and Biological Characterization of Fucoidan from Fucus vesiculosus Purified by Dye Affinity Chromatography. Mar Drugs.

[33] Thomsen S (1991). Pathologic analysis of photothermal and photomechanical effects of laser–tissue interactions. Photochem Photobiol.

[34] Luke GP, Yeager D, Emelianov SY (2012). Biomedical applications of photoacoustic imaging with exogenous contrast agents. Ann Biomed Eng.

[35] Jain PK, Lee KS, El-Sayed IH, El-Sayed MA (2006). Calculated absorption and scattering properties of gold nanoparticles of different size, shape, and composition: applications in biological imaging and biomedicine. J Phys Chem B.

[36] de la Zerda A, Bodapati S, Teed R, May SY, Tabakman SM, Liu Z, Khuri-Yakub BT, Chen X, Dai H, Gambhir SS (2012). Family of enhanced photoacoustic imaging agents for high-sensitivity and multiplexing studies in living mice. ACS Nano.

[37] De La Zerda A, Zavaleta C, Keren S, Vaithilingam S, Bodapati S, Liu Z, Levi J, Smith BR, Ma TJ, Oralkan O (2008). Carbon nanotubes as photoacoustic molecular imaging agents in living mice. Nat Nanotechnol.

[38] Yang K, Hu L, Ma X, Ye S, Cheng L, Shi X, Li C, Li Y, Liu Z (2012). Multimodal imaging guided photothermal therapy using functionalized graphene nanosheets anchored with magnetic nanoparticles. Adv Mater.

[39] Baker M (2010). Nanotechnology imaging probes: smaller and more stable. Nat Meth.

[40] Huang X, Tang S, Mu X, Dai Y, Chen G, Zhou Z, Ruan F, Yang Z, Zheng N (2011). Freestanding palladium nanosheets with plasmonic and catalytic properties. Nat Nanotechnol.

[41] Dorris A, Rucareanu S, Reven L, Barrett CJ, Lennox RB (2008). Preparation and characterization of polyelectrolyte-coated gold nanoparticles. Langmuir.

[42] Yue C, Liu P, Zheng M, Zhao P, Wang Y, Ma Y, Cai L (2013). IR-780 dye loaded tumor targeting theranostic nanoparticles for NIR imaging and photothermal therapy. Biomaterials.

[43] Peng J, Zhao L, Zhu X, Sun Y, Feng W, Gao Y, Wang L, Li F (2013). Hollow silica nanoparticles loaded with hydrophobic phthalocyanine for near-infrared photodynamic and photothermal combination therapy. Biomaterials.

[44] Xia L, Kong X, Liu X, Tu L, Zhang Y, Chang Y, Liu K, Shen D, Zhao H, Zhang H (2014). An upconversion nanoparticle–zinc phthalocyanine based nanophotosensitizer for photodynamic therapy. Biomaterials.

[45] Cheng L, Liu J, Gu X, Gong H, Shi X, Liu T, Wang C, Wang X, Liu G, Xing H (2014). PEGylated WS2 Nanosheets as a Multifunctional Theranostic Agent for *in vivo* Dual-Modal CT/Photoacoustic Imaging Guided Photothermal Therapy. Adv Mater.

[46] Kim SK (2013). Marine nutraceuticals: prospects and perspectives.

[47] Vo TS, Kim SK (2013). Fucoidans as a natural bioactive ingredient for functional foods. J Funct Foods.

[48] Lee H, Kim JS, Kim E (2012). Fucoidan from seaweed Fucus vesiculosus inhibits migration and invasion of human lung cancer cell via PI3K-Akt-mTOR pathways. PLoS One.

[49] Tacar O, Sriamornsak P, Dass CR (2013). Doxorubicin: an update on anticancer molecular action, toxicity and novel drug delivery systems. J Pharm Pharmacol.

[50] Markovic ZM, Harhaji-Trajkovic LM, Todorovic-Markovic BM, Kepić DP, Arsikin KM, Jovanović SP, Pantovic AC, Dramićanin MD, Trajkovic VS (2011). In vitro comparison of the photothermal anticancer activity of graphene nanoparticles and carbon nanotubes. Biomaterials.

[51] Lüpertz R, Wätjen W, Kahl R, Chovolou Y (2010). Dose-and time-dependent effects of doxorubicin on cytotoxicity, cell cycle and apoptotic cell death in human colon cancer cells. Toxicology.

[52] Zhao X, Chen Q, Liu W, Li Y, Tang H, Liu X, Yang X (2015). Codelivery of doxorubicin and curcumin with lipid nanoparticles results in improved efficacy of chemotherapy in liver cancer. Int J Nanomed.

[53] Yuan A, Wu J, Tang X, Zhao L, Xu F, Hu Y (2013). Application of near-infrared dyes for tumor imaging, photothermal, and photodynamic therapies. J Pharm Sci.

[54] Welch AJ, Van Gemert MJ (2011). Optical-thermal response of laser-irradiated tissue.

[55] Shao J, Griffin RJ, Galanzha EI, Kim JW, Koonce N, Webber J, Mustafa T, Biris AS, Nedosekin DA, Zharov VP (2013). Photothermal nanodrugs: potential of TNF-gold nanospheres for cancer theranostics. Sci Rep.

[56] Li JL, Wang L, Liu XY, Zhang ZP, Guo HC, Liu WM, Tang SH (2009). *In vitro* cancer cell imaging and therapy using transferrin-conjugated gold nanoparticles. Cancer Lett.

[57] Duan R, Zhou Z, Su G, Liu L, Guan M, Du B, Zhang Q (2014). Chitosan-coated Gold Nanorods for Cancer Therapy Combining Chemical and Photothermal Effects. Macromol Biosci.

[58] Prahl SA http://omlc.org/spectra/hemoglobin/.

[59] Holowka EP, Bhatia SK (2014). Drug Delivery: Materials Design and Clinical Perspective.

[60] Wong AD, Ye M, Ulmschneider MB, Searson PC (2015). Quantitative analysis of the enhanced permeation and retention (EPR) effect. PLoS One.

[61] Maeda H, Sawa T, Konno T (2001). Mechanism of tumor-targeted delivery of macromolecular drugs, including the EPR effect in solid tumor and clinical overview of the prototype polymeric drug SMANCS. J Control Release.

[62] Manivasagan P, Bharathiraja S, Santha Moorthy M, Oh YO, Song K, Seo H, Oh J (2017). Anti-EGFR antibody conjugation of fucoidan-coated gold nanorods as novel photothermal ablation agents for cancer therapy. ACS App Mat Interface.

[63] Nguyen VP, Oh J, Park S, Wook Kang H (2017). Feasibility of photoacoustic evaluations on dual-thermal treatment of *ex vivo* bladder tumors. J Biophotonics.

