# Supplementary material for: Doxorubicin-fucoidan-gold nanoparticles composite for dual-chemo-photothermal treatment on eye tumors
Source: Oncotarget. 2017 Dec 9;8(69):113719–33. doi: 10.18632/oncotarget.23092 (PMC5768358; doi:10.18632/oncotarget.23092)
Supplement: Supplementary file 1 [file oncotarget-08-113719-s001.pdf]

Doxorubicin-fucoidan-gold nanoparticles composite for dual-chemo-photothermal treatment on eye tumors

SUPPLEMENTARY MATERIALS

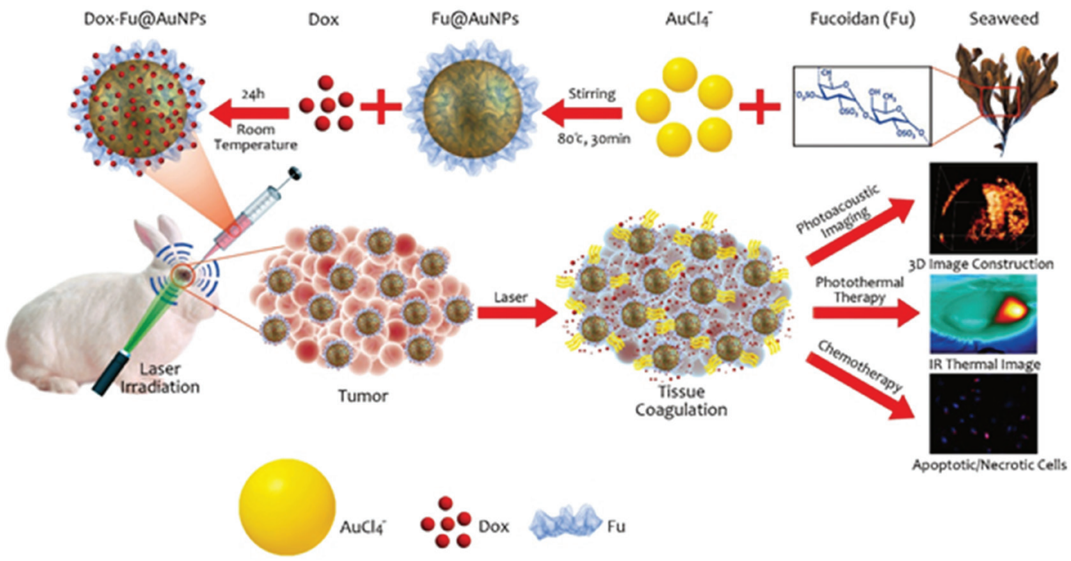

Supplementary Figure 1: Description of Fu and Dox chemical structures and synthetic procedure for biocompatible Dox-Fu@AuNPs.

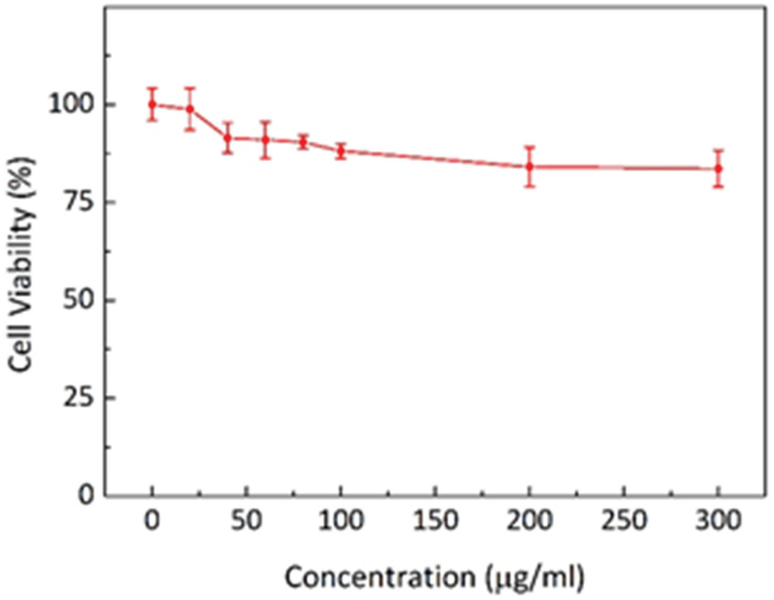

Supplementary Figure 2: Effect on Dox-Fu@AuNPs on Raw 264.7 macrophages at various concentrations for 24 h.

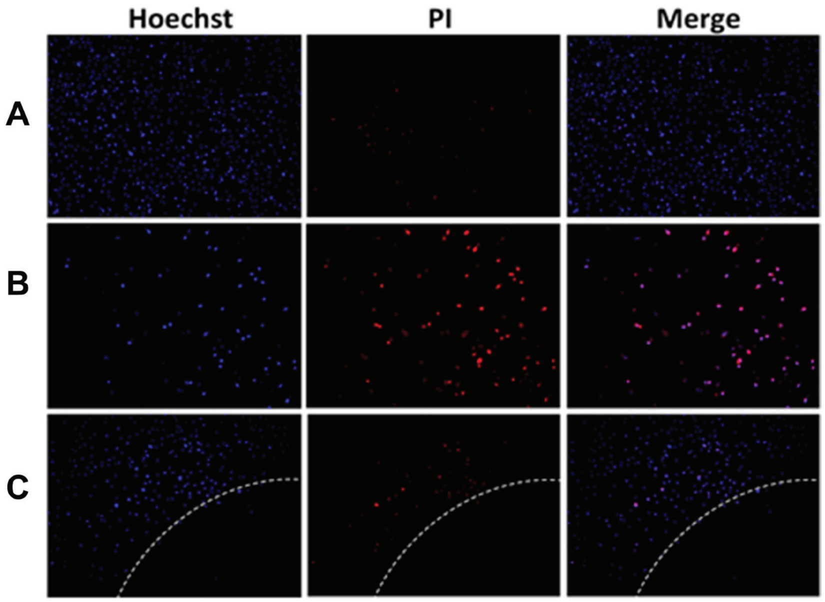

Supplementary Figure 3: Fluorescence microscope images of VX2 cells stained with Hoechst and PI after laser irradiation at different positions and staining. The fluorescence and bright field images of the cells were located: (**A**) outside, (**B**) inside, and (**C**) on the edge near the treated field (20×; bar = 50 μm).

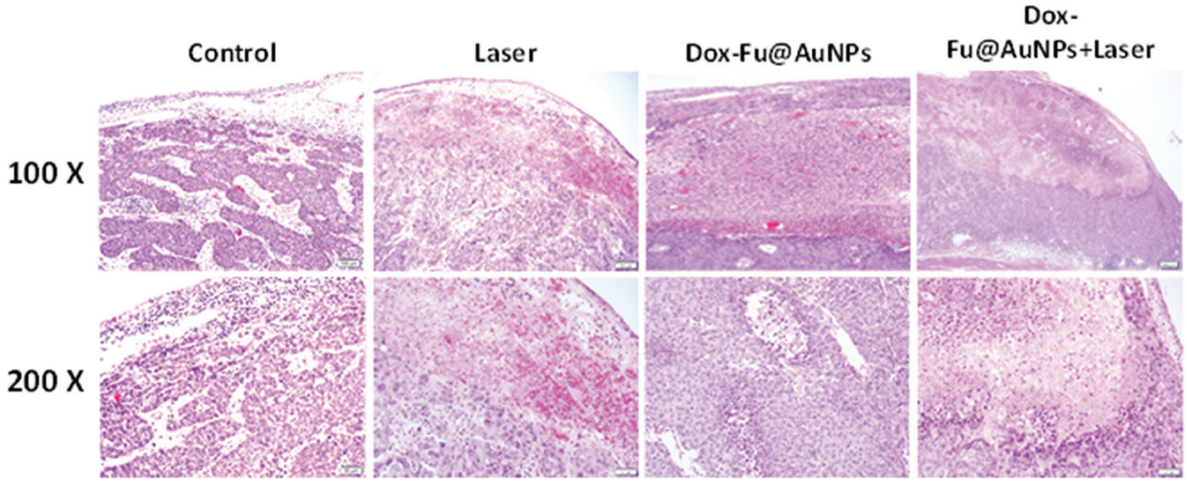

Supplementary Figure 4: Histology images of tumor tissues obtained from four different groups 14 days after each treatment: rabbits treated with saline (control), laser only, Dox-Fu@AuNPs only, and Dox-Fu@AuNPs with laser irradiation.
